# Mesenchymal stem/stromal cell quality control: validation of mixed lymphocyte reaction assay using flow cytometry according to ICH Q2(R1)

**DOI:** 10.1186/s13287-020-01947-6

**Published:** 2020-10-01

**Authors:** Tess Nicotra, Aurélie Desnos, Justine Halimi, Hélène Antonot, Loïc Reppel, Thomas Belmas, Alice Freton, Floriane Stranieri, Miryam Mebarki, Jérôme Larghero, Audrey Cras, Lionel Faivre

**Affiliations:** 1 AP-HP, Hôpital Saint-Louis, Unité de Thérapie Cellulaire, 75010 Paris, France; 2 Université Paris Descartes, Sorbonne Paris Cité, 75006 Paris, France; 3 CHRU de Nancy, Unité de Thérapie Cellulaire et banque de tissus, 54500 Vandœuvre-lès-Nancy, France; 4 CNRS, UMR 7365, 54500 Vandœuvre-lès-Nancy, France; 5 Université de Lorraine, 54000 Nancy, France; 6 Université de Paris, Inserm, U976 HIPI, F-75006 Paris, France; 7 Université Paris Diderot, Sorbonne Paris Cité, 75010 Paris, France; 8 Inserm UMR_S1140, Faculté de Pharmacie, 75006 Paris, France

**Keywords:** Mesenchymal stem/stromal cell, Lymphocyte proliferation, Biological assay, Potency assay, Quality control, Mixed lymphocyte reaction, Immune response modulation, Clinical trial, MSC manufacturing

## Abstract

**Background:**

Mesenchymal stem/stromal cells (MSC) have immunomodulatory properties, studied in a wide range of diseases. Validated quality controls must confirm this activity in the context of clinical trials. This study presents a method’s validation, assessing MSC’s ability to inhibit lymphocyte proliferation, according to the ICH Q2 standard.

**Methods:**

MSC were co-cultured with CellTrace™ Violet-labeled Peripheral blood mononuclear cells (PBMC) coming from a bank of ten donors, at seven different ratios for 7 days. Cell trace violet PBMC bank was validated in parallel. Flow cytometry analysis was used to obtain the division percentage of T cells. The percentage of inhibition of lymphocyte proliferation by MSC, for each ratio X, was calculated using the formula: Ratio × percentage of inhibition = (control percentage of division—ratio × percentage of division)/control percentage of division. The inhibition percentage of lymphocyte proliferation function of co-culture ratios was represented in a line graph. The corresponding area under the curve was calculated, representing MSC’s ability to inhibit lymphocyte proliferation.

**Results:**

Two cell trace violet PBMC banks were compared for bank validation. When compared using four different MSC samples coming each from a different donor, their area under the curve did not show any statistical differences and were correlated. Moreover, the stability of one cell trace violet PBMC bank was confirmed up to 509 days of storage.

Analytical parameters were investigated for method validation. Analysis of repeatability and reproducibility respectively showed a standard deviation of 6.1% and 4.6%. The assay was robust regarding PBMC, as no statistical differences were found between inhibitory activities when testing three adjacent concentrations of PBMC. Still, attention is needed on MSC quantity as it can influence results. Linearity was evaluated: the percentage of inhibition of lymphocyte proliferation function of co-culture ratios was linear on the exploited range. Finally, the assay measurement range allowed to differentiate MSC presenting different inhibition activities.

**Conclusion:**

This quantification method displayed low analytical variability and no inter-bank variability of PBMC. However, MSC quantification should be checked before co-culture to reduce variability. Therefore, it could be used for the qualification of MSC batches’ immunomodulatory activity.

## Background

Mesenchymal stem/stromal cells (MSC) are multipotent cells found in several tissues [1]. They have a strong potential to modulate the response of innate and adaptive immune cells, explaining the current enthusiasm for their use in many clinical trials of auto-immune diseases [2].

During the production of clinical MSC batches, according to the Good Manufacturing Practices (GMP), mandatory quality controls are carried out to assess their identity, purity, sterility, and potency. The potency assessment of MSC should be based on their mechanism of action in the targeted disorder, to reflect their biological activity after administration in patients. However, this task faces two challenges. First, described MSC mechanisms of action are numerous and complex [2]. Second, a correlation between their biological profile and the clinical outcome is still difficult to establish.

There is an extensive literature concerning the analysis of the immunomodulatory activities of MSC. Many types of assays are presented with different configurations, depending on the type of the evaluated immune cells. Parameters such as stimuli, readout, and time of analysis may vary among assays [3, 4].

Since in our unit, we produce MSC for clinical trials of systemic sclerosis and systemic lupus erythematosus, we are particularly interested in these two pathologies. For this reason, we used the mixed lymphocyte reaction (MLR) potency assay described by the Experimental and Clinical Cell Therapy Institute of Salzburg in Austria [5, 6] to evaluate our cells. The only change made concerns the fluorochrome used which was CellTrace™ Violet (CTV) instead of CFSE. This method has the advantage of evaluating the percentage of inhibition (PI) of lymphocyte proliferation by MSC, mimicking the biological context of these diseases. The low variability of this MLR is among critical criteria which make it analytically reliable according to the guidelines of the International Conference on Harmonization (ICH) Q2 (R1): Validation of Analytical Procedures: Text and Methodology. Here, we present an analytical validation of this MLR to demonstrate its reliability for clinical use, according to GMP standards. As stated in the ICH Q2 (R1) guidelines, variability is quantified to determine if it is analytically relevant for the functional characterization of MSC.

## Methods

### Isolation of PBMC and MSC

All samples collected from healthy adult donors were obtained after a written and informed consent, following the Helsinki’s Declaration and Health Authorities (French Biomedical Agency, Paris, France). Approval was obtained for all human cell sample collections and studies from the Comité de Protection des Personnes Ile de France IV (IRB00003835). For each peripheral blood mononuclear cells (PBMC) bank, ten residues of buffy coats containing leukocytes were purchased from the Etablissement Français du Sang (EFS Bichat, Paris). PBMC were isolated by density gradient centrifugation using Lymphocytes Separation Medium (Eurobio, Cat No: CMSMSL01-01), as described previously [5, 6]. All PBMC were collected in phosphate-buffered saline (PBS, Eurobio, Cat No: CS1PBS01-01) with 2 mM of ethylenediaminetetraacetic acid (EDTA, Sigma, Cat No: E5134) and washed twice. Equal numbers of PBMC from each buffy coat were pooled together after counting. The amount of pooled cells differed from one bank to another since the number of collected PBMC was different for each donor.

MSC were isolated from the human bone marrow (BM-MSC)**.** Bone marrow cells were plated in polystyrene flasks at 1 × 10^5^ total nucleated cells/cm^2^, in minimum essential medium α (MEM α) with GlutaMAX™ Supplement, no nucleosides medium (Life Technologies, Gibco™, Cat No: 32561094) supplemented with 10% of fetal bovine serum (FBS, Dutscher, Cat No: S1810-500), 10 ng/ml of fibroblast growth factor (FGF, R&D Systems®, Cat No: 233-FB-500), and 1% of antibiotic/antimycotic (Thermo Fisher Scientific, Gibco™, Cat No: 15240062). Cells were cultured at 37 °C and 5% CO_2._ After 24 to 48 h, non-adherent cells were discarded. Fresh medium was added and renewed twice a week. At 70–80% confluence, adherent cells were trypsinized, washed, and seeded at 1 × 10^4^ cells/cm^2^. In all experiments, BM-MSC were used at passages 2–5, after maximum one freezing/thawing at an early passage. MSC were also phenotypically characterized by flow cytometry (Navios, Beckman Coulter) based on the expression of surface markers CD45−, CD73+, CD90+, and CD105+, according to the recommendations of the International Society for Cell & Gene Therapy (ISCT) [7]. Antibodies used for the staining were all purchased from BD Pharmingen™: anti-CD45-FITC (Cat No: 555482), anti-CD73-PE (Cat No: 550257), anti-CD90-FITC (Cat No: 555595), and anti-CD105-PE (Cat No: 560839). For isotype staining, antibodies IgG1k-FITC (Cat No: 555748) and isotype IgG1k-PE (Cat No: 555749) were used.

### CTV labeling and bank constitution

**Fig. 1:**
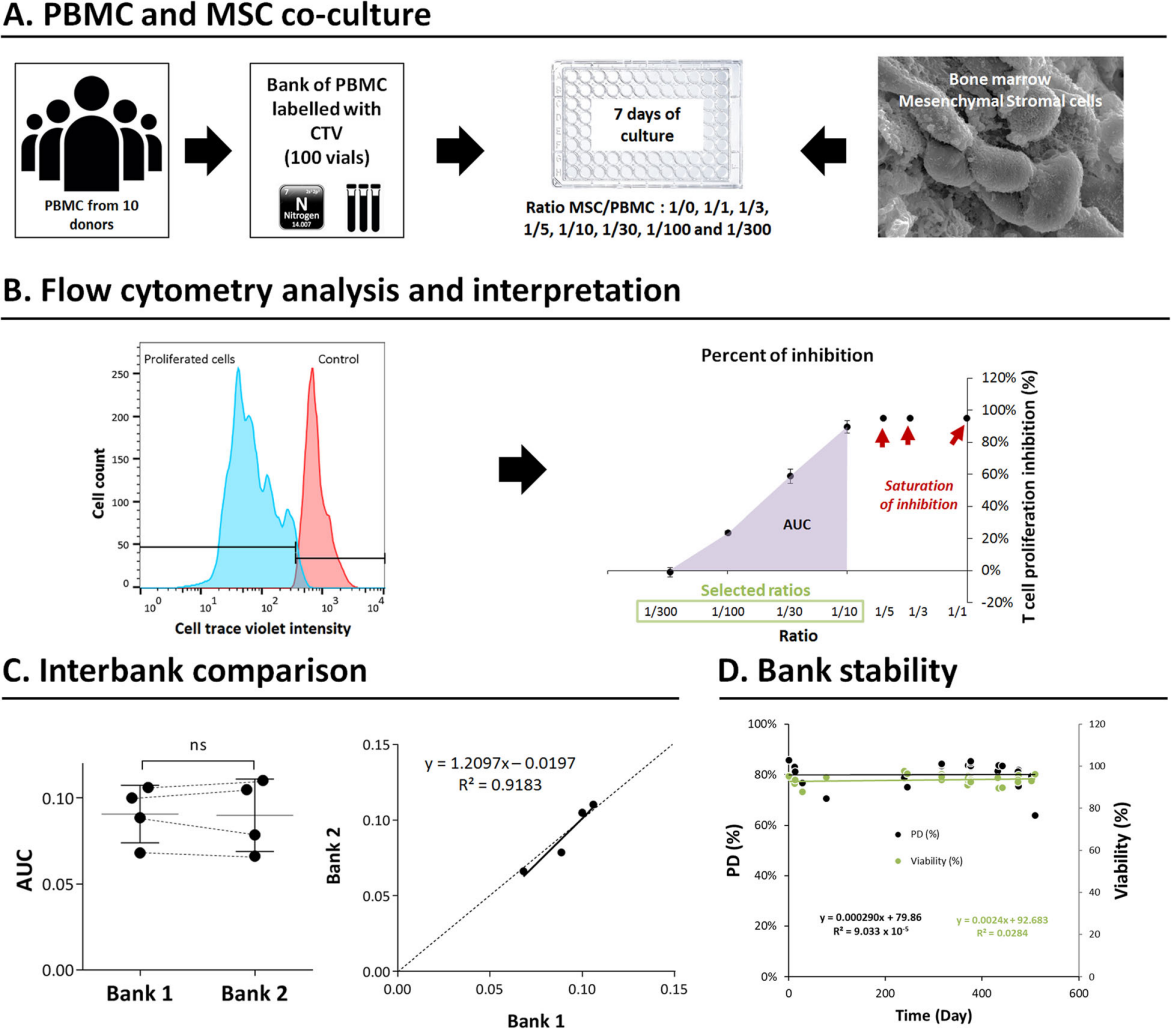
Schematic description of MLR assay: PBMC and MSC co-culture, analysis, interpretation, and CTV-PBMC bank validation. **a** A pool of PBMC from 10 donors was labeled with CTV, frozen, and stored in liquid nitrogen in 100 vials. During 7 days, BM-MSC and CTV-PBMC were co-cultured at different ratios (BM-MSC/CTV-PBMC): 1/1, 1/3, 1/5, 1/10, 1/30, 1/100, and 1/300. **b** After culture, CTV-PBMC were collected and analyzed by flow cytometry to determine lymphocytes’ PD. The AUC of a linear section of the PBMC/MSC ratio-dependent PI of the lymphocyte proliferation curve was calculated. **c** A comparison between two CTV-PBMC banks was proceeded using BM-MSC from four donors. AUCs of the selected ratios of the two banks were non-statistically different (*p* value > 0.9999; two-tailed Wilcoxon matched-pairs signed-rank test; *n* = 4) and correlated (*p* value = 0.0417; two-tailed Pearson’s correlation test). Equation is [Bank 2] = 1.208 × [bank 1] – 0.01958 with a regression coefficient: *R* squared = 0.9188. **d** The-PBMC bank’s stability was tested for 509 days without a decrease in the lymphocytes’ PD or CD45+ cell viability after culture. The slopes of the curves representing either the PD or CD45+ viability over time are not significantly different from zero (Fisher test, *p* value = 0.9575 and 0.9355, respectively). The regression coefficient for these curves obtained is *R* squared = 9.033E−5 and 0.0284, respectively

**Fig. 2:**
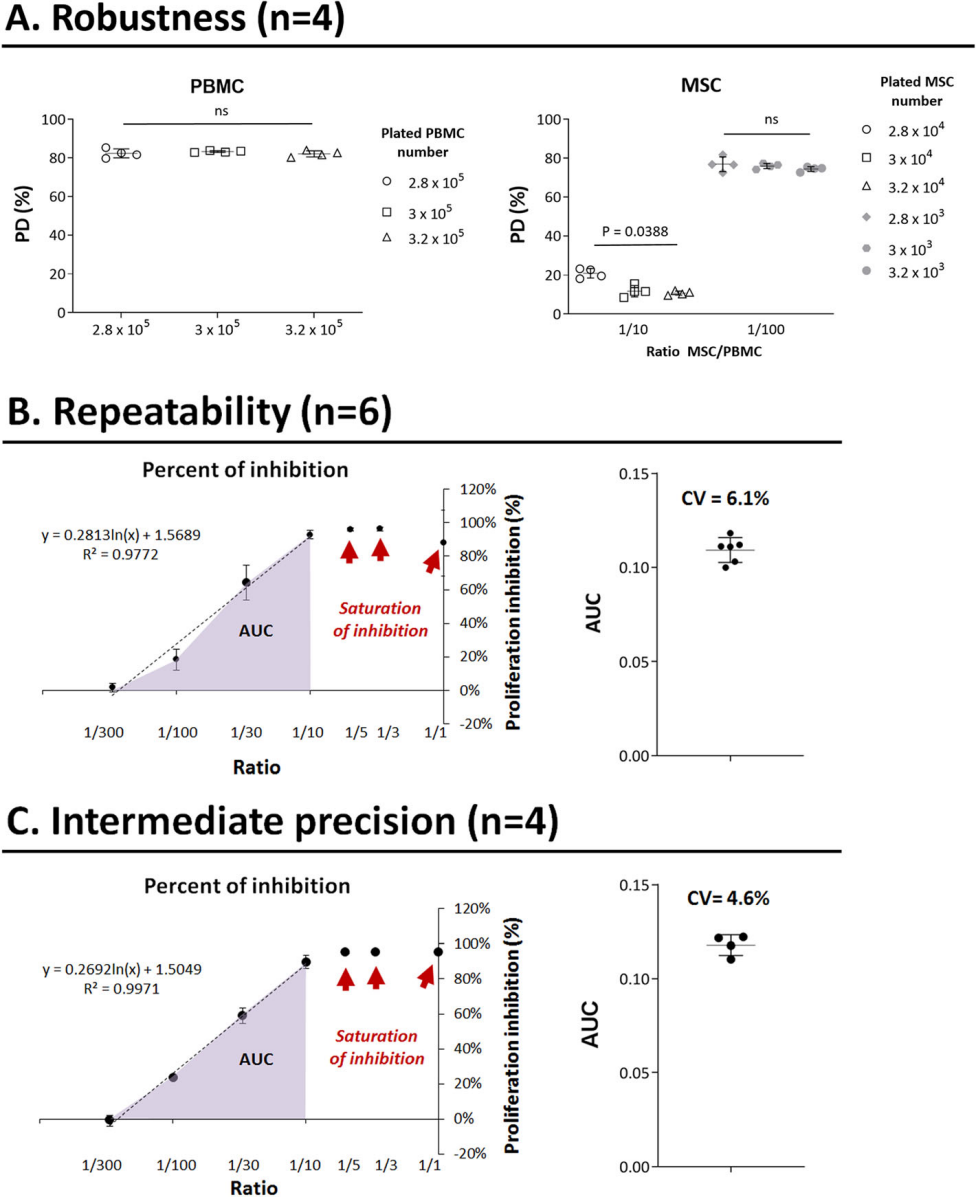
Results of robustness, repeatability, and intermediate precision parameters. **a** Robustness was tested with small but voluntary variations on CTV-PBMC and MSC quantity parameters. For CTV-PBMC robustness, two quantities were tested around 3 × 10^5^: 2.8 × 10^5^ and 3.2 × 10^5^. CTV-PBMC were seeded alone in the same conditions as described. Variations did not show any statistical difference between CTV-PBMC groups (*p* value = 0.4723, Friedman test; *n* = 4). For MSC quantity, two PBMC/MSC ratios were tested to assess MSC quantity’s robustness, 1/10, and 1/100. Variations did not show any statistical difference between groups for the 1/100 ratio (*p* value = 0.4724, Friedman test, *n* = 4) but a statistical difference for the 1/10 ratio (*p* value = 0.0388, Friedman test, *n* = 4). The linear portion of the curve has a regression coefficient R squared = 0.9772. **b** Repeatability of the assay was performed by repeating six times the entire MLR method on the same MSC sample from a single donor by the same operator the same day. CTV-PBMC percent of inhibition were linear on ratios 1/10, 1/30, 1/10, and 1/300. AUC on these points was showed a CV = 6.1%. The linear portion of the curve has a regression coefficient *R* squared = 0.9972. **c** Intermediate precision was performed by repeating four times the entire MLR method on the same MSC sample from a single donor by four operators the same day. CTV-PBMC percent of inhibition were linear on ratios 1/10, 1/30, 1/10, and 1/300. The calculation of CV for the four analyses was 4.6%

**Fig. 3:**
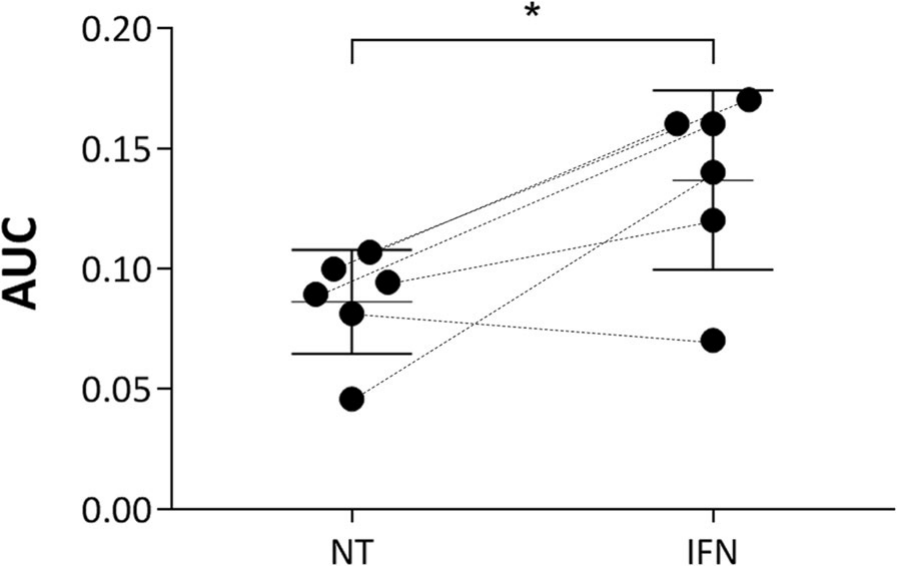
MLR results with MSC primed or not with IFNγ. Six batches of MSC were tested after being primed for 72 h with IFNγ (IFN) or not (NT) using MLR assay to determine if this assay can discriminate a more potent MSC batch or not. The two groups were significantly different from one and another, with an increase of the response in the IFNγ primed-MSC group (*p* value = 0.0313, two-tailed Wilcoxon matched-pairs signed-rank test; *n* = 6)

Pooled PBMC were labeled using the CTV Cell Proliferation Kit (Invitrogen™, Cat No: C34557). 2 × 10^7^ PBMC/ml were incubated with 2.5 μM of CTV, for 20 min at 37 °C. To neutralize the unbounded CTV, labeled PBMC were incubated for 5 min with an equal volume of Roswell Park Memorial Institute (RPMI) Medium 1640, GlutaMAX™ Supplement, HEPES (Gibco™, Cat No: 72400-013) at 6 g/L, supplemented with 10% of human A/B serum (Eurobio, Cat No: CAEHUM01-0 U), 1% of Antibiotic-Antimycotic (Amphotericin B, Penicillin, Streptomycin, Gibco™ by Life Technologies, Cat No: 15240-062), and 10 UI/ml of Heparin (PanPharma, Cat No: 552050-8). The staining and the neutralization were done protected from light. After rinsing with PBS/EDTA, cells were counted using an automatic hematopoietic counter (AcT5 Diff., Beckman Coulter). CD3/CD45/7-AAD staining was done on a sample of labeled cells and analyzed by flow cytometry (see details in section “Flow Cytometry Analysis”). CTV-PBMC (i.e., PBMC bank labeled with CTV) were frozen in FBS with 10% dimethyl sulfoxide (DMSO) at − 80 °C, in Nalgene® Mr. Frosty. One hundred aliquots containing between 2.5 × 10^7^ and 3.5 × 10^7^ CTV-PBMC/ml were prepared to constitute the CTV-PBMC bank. Twenty-hours later, aliquots were transferred in liquid nitrogen for long term storage. Two CTV-PBMC banks (i.e., banks 1 and 2) were constituted and compared for the CTV-PBMC bank validation (Fig. 1c). Only bank 1 was used for the co-culture experiments (Figs. 2 and 3). The stability of bank 1 was assessed up to 509 days (Fig. 1d).

### Co-culture of CTV-PBMC and MSC

MLR was used as described by Ketterl et al. with the following changes to assess the immunomodulatory effect of BM-MSC (Fig. 1a) [5]. For co-culture, the following ratios of MSC/CTV-PBMC were tested: 0/1 (control), 1/1, 1/3, 1/5, 1/10, 1/30, 1/100, and 1/300. MSC number added per well varied upon ratios whereas CTV-PBMC number per well was constant (i.e., 3 × 10^5^ CTV-PBMC per well). One day before starting the co-culture, MSC were plated in duplicates in a flat-bottomed 96-well plate, according to the ratios to be tested (i.e., for each ratio, number of MSC per well in 200 μl: 0, 3 × 10^5^, 1 × 10^5^, 6 × 10^4^, 3 × 10^4^, 1 × 10^4^, 3 × 10^3^, 1 × 10^3^). On the day of the co-culture, CTV-PBMC were thawed at 37 °C in the co-culture medium (see below). Thawed CTV-PBMC were washed with the medium and counted. Quality control was done on a sample of labeled cells and analyzed by flow cytometry. Cell viability and CTV fluorescence intensity were compared to before freezing. Thawing yield was calculated according to the following formula: (lymphocyte number after thawing/frozen lymphocyte number)*100. The cell suspension was prepared to obtain 3 × 10^5^ PBMC in 250 μl of medium per well, corresponding to 5 × 10^6^ PBMC in total (see section “CTV labeling and bank constitution” for medium composition). PBMC were added to the flat-bottomed 96-well plate previously seeded with MSC. Cells were incubated for 7 days at 37 °C, with 5% CO_2_. At day 4, 50 μl of the medium was added to each well, and cell proliferation was observed with a microscope. On day 7, CTV-PBMC were collected from the co-culture wells by a gentle resuspension without collecting MSC as they are adherent, and duplicates were pooled together. After washing with PBS, quality control was done on each ratio and analyzed by flow cytometry.

### IFNγ treatment of MSC

MSC were treated with IFNγ, known to activate their immunomodulatory properties [8]. MSC at 80% confluence were treated in vitro with recombinant human IFNγ protein (R&D Systems®, Cat No: 285-IF-100) at 10 ng/ml for 48 h. After treatment, MSC were collected and counted. Different quantities of MSC were plated to match each co-culture ratio, in a flat-bottomed 96-well plate, with medium containing IFNγ. The next day, co-culture began, with thawed CTV-PBMC, as described above. In total, MSC were pre-treated with IFNγ for 72 h.

### Flow cytometry analysis

CD3/CD45/7-AAD staining was realized on PBMC for 15 min at 4 °C with 5 μl of antibodies: anti-CD3 PE (BD Biosciences, Cat No: 345765), anti-CD45 FITC (BD Biosciences, Cat No: 345808), and 7-AAD viability dye (Beckman Coulter, Cat No: A07704). After washing with PBS, cell staining was evaluated using the Navios cytometer (Beckman Coulter). Flow cytometry results were analyzed with FlowJo™ v10 software. T cell proliferation was calculated after excluding cell doublets, dead cells, and selecting CD3+/CD45+/viable cells, using the measure of CTV fluorescence intensity (Fig. 1b, left panel). Percentage of division (PD) was determined among viable CD3+/CD45+ cells by using CTV fluorescence intensity to separate proliferated and non-proliferated cells (i.e., cells with low- or high CTV-fluorescence intensity, respectively). PD was calculated using the following formula = (number of proliferated cells)/[(number of proliferated cells) + (number of non-proliferated cells)]. For each ratio of co-cultured CTV-PBMC/MSC, the PI of lymphocyte proliferation was calculated using the following formula = (PD of control—PD of ratio)/(PD of control). The control sample corresponds to the ratio of MSC/CTV-PBMC 0/1. Then, the PI of T cell proliferation function of co-culture ratios was represented in a line graph (Fig. 1b, right panel). The area under the curve (AUC) of the described line graph was calculated only for the selected ratios (i.e., ratios for which the curve is linear) using the trapezoidal rule as previously described [9] (Fig. 1b, right panel). This AUC represents MSC’s ability to inhibit lymphocyte proliferation, assessing their immunomodulatory activity. The ratios for which we observe a saturation of the PI of lymphocyte proliferation (i.e., ratios 1/5, 1/3, and 1/1 in Fig. 1b) were not selected for AUC calculation.

### Validation of the analytical method

#### Analytical parameters investigated

- **Validation of CTV-PBMC bank** by (1) comparing two banks (bank 1 and bank 2) of CTV-PBMC, each coming from ten different donors. To this aim, a complete analysis of the two banks was done. The same four different MSC samples, each coming from a different donor, were tested using either bank 1 or bank 2, and results were compared. (2) By studying bank stability over time. The PD of control (MSC/CTV-PBMC ratio: 0/1) and CD45+ cell viability after the culture of bank 1 was tested over 509 days. All the experiments were carried out in the meantime.
- **Robustness** is the ability of a method to withstand small but deliberate variations in its critical parameters. It indicates the method of reliability under normal conditions of use. Two parameters were chosen for deliberate variations: CTV-PBMC quantity and MSC quantity used for the co-culture. Three adjacent concentrations of plated CTV-PBMC were tested to observe the variation effect in the quantity of plated CTV-PBMC. 2.8 × 10^5^, 3 × 10^5^ (quantity used as described in Salzburg’s assay protocol, called “normal quantity” in this paper), and 3.2 × 10^5^ CTV-PBMC were plated without MSC, in a flat-bottomed 96-well plate. To assess the impact of the variation of MSC quantity on the inhibition of CTV-PBMC proliferation, two co-culture ratios of MSC/CTV-PBMC were tested. The ratio 1/10 was tested either with 2.8 × 10^4^, 3 × 10^4^ (normal quantity), or 3.2 × 10^4^ MSC, co-cultured in each case with the same number of CTV-PBMC (i.e., 3 × 10^5^ CTV-PBMC). The second tested ratio (*n* = 4 duplicates in one experiment) was 1/100 for which 2.8 × 10^3^, 3 × 10^3^ (normal quantity), or 3.2 × 10^3^ MSC were co-cultured with 3 × 10^5^ PBMC. The choice of an interval of 6.7% is related to the variability observed during the assay’s pipetting and cell counting steps.
- **Repeatability** indicates the fidelity of the method assessed under identical operating conditions in a short period. It is also called the intra-assay precision. Six successive analyses (*n* = 6) were done on the same MSC sample from a single donor. AUC of the percentage of inhibitory activity of all co-culture ratios (except ratios giving saturation of inhibition) was calculated.
- **Intermediate precision** shows the intra-laboratory variability. For example, different days, different operators, or different equipment are tested. It is usually applied to the standardization of the methodology. Four different analyses were repeated by four different operators (*n* = 4) on the same MSC sample from a single donor. AUC of the percentage of the inhibitory activity of all selected co-culture ratios was calculated.
- **Linearity of the method** was determined. The regression coefficient of the linear portion of the percentage of the inhibitory activity of co-culture ratios was calculated using GraphPad Prism version 6.04 for Windows, GraphPad Software, La Jolla California USA, www.graphpad.com.
- **Measurement range**: we analyzed the ability of the assay to distinguish MSC presenting different ranges of inhibitory activity. MSC were treated or not with IFNγ which is known to activate their immunomodulatory properties [8]. The objective of this analysis is to check the ability of the assay to distinguish different ranges of inhibition property, i.e., between untreated and IFNγ activated MSC.

### Statistical analysis

Values are expressed as follows: mean ± standard deviation (SD). Coefficients of variation (CV) were also calculated to assess the dispersion of results. A value of less than 10% for the CV is desired, corresponding to the usual limit value when little analytical validation data exist, according to the ISO 15189 standard. For repeated measures, statistical analyses used were two-tailed Wilcoxon matched-pairs signed-rank test, given that our data are non-normally distributed. For robustness, the Friedman test was used for statistical analyses. This test was chosen because it allows the comparison of non-parametric-matched datasets, which is the case here. Pearson’s correlation test was used for bank validation to assess the correlation between two CTV-PBMC banks. To evaluate bank stability over time, we used the Fisher test. It allows determining, for the graph representing the CTV-PBMC PD function of the number of days after the bank constitution, if the slope is significantly different from zero. The bank cannot be considered stable if the slope of the graph representing the lymphocyte PD function of the number of days is different from zero. Indeed, if the slope was different from zero (non-horizontal line), it would mean the lymphocyte PD of CTV-PBMC samples coming from the same batch would not be consistent over time. Values from two different groups were considered significantly different when *p* value < 0.05. All statistical tests were done using the GraphPad PRISM® version 6.02 software.

All data is accessible by following the attached link: 10.17632/btpn7s2bff.1#file-ccd44c21-dcf1-40b0-b137-55b6b7eacabf.

## Results

### MSC isolation

BM-MSC were isolated from six healthy donors. Their phenotype was analyzed by flow cytometry. Surface markers CD45, CD105, CD90, and CD73 were assessed. Results showed that all samples were CD45−; CD90+, with a mean ± SD of 99.40 ± 0.30% positive cells; CD73+, with a mean of 99.07 ± 0.64% positive cells; and CD105+, with a mean of 98.80 ± 0.72% positive cells. The average donor age was 20.5 ± 21.3 years, min. of 3 and max. of 57 years (Table 1).

**Table 1:** MSC phenotypic characterization, donor age, and passage

MSC donor	Phenotypic characterization (%)				Age (years)	Passage
	CD90	CD73	CD105	CD45		
1	99.4	99.2	99.3	0.0	3	5
2	99.8	99.8	99.7	0.0	8	4
3	99.6	99.6	98.0	0.0	4	5
4	98.9	99.2	97.9	0.0	34	3
5	99.4	98.2	98.8	0.0	17	4
6	99.3	98.4	99.1	0.0	57	5

### Bank validation

PBMC from 10 random healthy donors were pooled to constitute the CTV-PBMC banks 1 and 2. To obtain a homogeneous pool, in order to mimic physiological immune mechanisms and to reduce inter-individual variability, equal numbers of PBMC harvested from each donor were added. Thus, before pooling, we adjusted the number of PBMC from each donor to the lowest number of PBMC collected among donors. For bank 1, we pooled 3.333 × 10^8^ PBMC, and for bank 2, 3.120 × 10^8^ PBMC from each donor. After labeling and washing, we obtained in total 2.6 × 10^9^ and 2.8 × 10^9^ CTV-PBMC and froze 96 and 95 aliquots, containing 2.70 × 10^7^ or 2.90 × 10^7^ cells each, for bank 1 and bank 2, respectively. There was a mean of 18.21 ± 1.69 × 10^6^ white blood cells in total per aliquot, after thawing aliquots (*n* = 7) of bank 1. The composition of bank 1 (*n* = 7) was 14.10 ± 4.10% of neutrophils, 58.86 ± 2.79% of lymphocytes, and 22.54 ± 3.74% of monocytes. There was a mean of 10.66 ± 1.32 × 10^6^ white blood cells in total per aliquot after thawing aliquots (*n* = 8) of bank 2. Bank 2 (*n* = 8) was composed of 8.6 ± 2.06% of neutrophils, 56.33 ± 3.16% of lymphocytes, and 33.15 ± 3.81% of monocytes.

The comparison between the two CTV-PBMC banks (i.e., bank 1 and bank 2), proceeded on four BM-MSC samples, each coming from a different donor (*n* = 4), is shown in Fig. 1c. The graph on the left displays statistical analysis of the AUC calculated from the graph representing the PI lymphocyte proliferation function of the BM-MSC/PBMC ratios of co-culture (from 1/300 to 1/10), tested for the four BM-MSC samples. Each line represents the comparison of the AUC of one of the four BM-MSC samples. Results of bank validation reveal no statistical difference between the AUC’s mean obtained for the four MSC samples tested either with bank 1 or bank 2 (*p* value > 0.9999; two-tailed Wilcoxon matched-pairs signed-rank test, *n* = 4).

The graph on the right displays the correlation curve between AUC obtained either with bank 1 or bank 2. AUC of the selected co-culture ratios of banks 1 and 2 was correlated (*p* value = 0.0417; two-tailed Pearson’s correlation test). The correlation curve is linear and close to the regression curve. Its equation is: [Bank 2] = 1.208 × [bank 1] – 0.01958 with a regression coefficient: *R* squared = 0.9188, close to 1. There is non-statistical difference between the two banks.

Moreover, analysis of the PD and CD45+ cell viability after culture of bank 1 CTV-PBMC samples, stored in liquid nitrogen, and assessed over 509 days (Fig. 1d) showed excellent stability over time. Indeed, the slope of the curve, representing the PD over time was not significantly different from zero (Fisher test, *p* value = 0.9575). The regression coefficient for this curve obtained with GraphPad PRISM® was *R* squared = 9.033E−5, which is very close to 0, also showing that the PD was stable over time. Regarding CD45+ cell viability after culture over time, the slope of the curve was not significantly different from zero (Fisher test, *p* value = 0.9355). The regression coefficient was close to 0 (*R* squared = 0.0284) showing that the viability after culture was stable over time.

### Robustness

Two parameters were deliberately modified to analyze the robustness of the method (Fig. 2a). First, three densities of plated CTV-PBMC/well were tested: 2.8 × 10^5^, 3 × 10^5^ (control quantity, normally used for the assay), and 3.2 × 10^5^ (Fig. 2a left panel). Results showed that CTV-PBMC PD mean was 81.4 ± 2.3% for 2.8 × 10^5^ PBMC, 83.3 ± 0.5% for 3 × 10^5^ PBMC, and 82.2 ± 1.6% for 3.2 × 10^5^ PBMC. Statistical analysis showed no significant difference between the CTV-PBMC tested densities (*p* value = 0.4723, Friedman test, *n* = 4).

The second parameter to be modified was MSC quantity used for co-culture (Fig. 2a right panel). As previously, three concentrations of plated MSC, at the co-culture ratio of 1/10 with PBMC, were tested: 2.8 × 10^4^, 3 × 10^4^ (control quantity), and 3.2 × 10^4^. Results showed that the mean of inhibition percentage is 20.2 ± 2.4%, 11.8 ± 2.9%, and 10.8 ± 1.0% for MSC plated at densities of 2.8 × 10^4^, 3 × 10^4^, and 3.2 × 10^4^ cells/well, respectively. Statistical analysis, for 1/10 ratio, demonstrated a statistically significant difference between groups (*p* value = 0.0388, Friedman test, *n* = 4). Regarding 1/100 co-culture ratio, inhibition percentage means were 76.9 ± 3.8%, 76.0 ± 1.4%, and 74.5 ± 1.2%, for MSC plated numbers of 2.8 × 10^3^, 3 × 10^3^, and 3.2 × 10^3^, respectively. There was no significant statistical difference between groups for the 1/100 co-culture ratio (*p* value = 0.4724, Friedman test, *n* = 4).

### Repeatability

Six successive co-culture analyses (*n* = 6) were done on the same MSC sample from a single donor to analyze the method’s repeatability (Fig. 2b). Then, AUC of the lymphocyte proliferation’s PI of all selected co-culture ratios (1/10 to 1/300) was calculated (Fig. 2b, left panel). The mean of the AUC was 0.109 ± 0.007, with a CV of 6.1% (Fig. 2b, right panel).

### Intermediate precision

Intermediate precision was also assessed by doing four co-culture analyses of the same MSC sample from a single donor, done by four different operators from the same laboratory (*n* = 4). AUC of lymphocyte proliferation’s PI of all co-culture ratios was calculated (Fig. 2c), with a mean of 0.105 ± 0.004, with a CV of 3.4% (Fig. 2c, right panel).

### Linearity

To assess the linearity of the method, we considered the curve representing the lymphocyte proliferation’s PI function of the co-culture ratios. The linearity of the selected portion of the curve (ratios 1/300 to 1/10) was tested (Fig. 2b, c). The regression curve was obtained and its equation was calculated: *y* = 0.2813 × ln[x] + 1.5689, and *y* = 0.2692 × ln[x) + 1.5049 (left panels of Fig. 2b, c, respectively). The linear regression coefficient was also calculated for both and was close to 1: *R* squared = 0.9772 and *R* squared = 0.9971 (left panels of Fig. 2b, c, respectively). Thus, the curve was considered linear from co-culture ratios 1/300 to 1/10.

### Ability of the assay to distinguish MSC presenting different ranges of inhibitory activity

The ability of the assay to distinguish MSC that display different ranges of inhibition activity (i.e., MSC stimulated or not with IFNγ) was also tested (Fig. 3). The AUC of the curve representing lymphocyte PI function of co-culture ratios was calculated on the linear portion of the curve to measure the immunomodulatory activity of both MSC groups. The higher the AUC, the higher the inhibition effect of MSC on lymphocyte proliferation is. The non-linear portion of the curve corresponds to a saturation of the inhibition on lymphocyte PD observed for high co-culture ratios (i.e., 1/1, 1/3, and 1/5). For this reason, we did not use this part of the curve for AUC calculation. AUC mean for the untreated group was 0.086 ± 0.022 and 0.136 ± 0.036 for IFNγ treated MSC. Results showed a statistically significant difference between the mean of AUC obtained for IFNγ-treated MSC, and the one obtained for untreated MSC (*p* value = 0.0313, two-tailed Wilcoxon matched-pairs signed-rank test, *n* = 6). Thus, this method seems to be able to distinguish MSC presenting different ranges of inhibition activity.

## Discussion

This study presents the validation of a quantification method, measuring the immunomodulatory properties of MSC on T cells with the MLR method, according to the ICH Q2 (R1) guideline. The chosen method was previously developed by the team of the Experimental and Clinical Cell Therapy Institute of Salzburg in Austria, except for the fluorochrome used which has been replaced by CTV. It consists of following the proliferation of T cells among CTV-PBMC co-cultured with MSC at different ratios, by flow cytometry, and over time [5, 6]. Here, the analysis of the data was improved. Previously, MSC’s effect on T cell PD was assessed by measuring the T cell PD for each co-culture ratio [5]. We considered the curve representing the PI of T cell function of co-culture ratios (BM-MSC/CTV-PBMC ratios 1/10, 1/30, 1/100, and 1/300). Here, we calculated the AUC on the linear portion of this curve, as described by Klinker et al. [9]. The choice of these ratios seems dependent on the origin of the MSC. To apply this method on umbilical cord-derived MSC, which present the most potent immunomodulatory activity [10], analysis of other ratios (data not shown) is required. It allows us to take into account the results of all ratios and to analyze only the results of the linear part of the ratio/inhibition percentage curve. Indeed, the non-linear portion of the curve corresponds to a saturation of the lymphocyte proliferation’s PI observed for high co-culture ratios (i.e., 1/1, 1/3, and 1/5). We did not use this part of the curve for AUC calculation as it would give a biased result. Finally, it leads to less variability and a higher representativity of MSC immunomodulatory response, compared to the result obtained by a single co-culture ratio.

Our method is a potency assay for producing clinical MSC batches, indicated for the treatment of auto-immune diseases. As the mechanism of action involved in these diseases is, inter alia, mediated by a T cell proliferation [11], this method assesses the effect of MSC on T cells. Thus, this method will be useful for the qualification of GMP manufactured MSC when the pathophysiology involves T lymphocytes. This MLR potency assay has advantages compared to other existing ones [3]. First, it mimics the physiological antigen presentation to T cells in disorders involving T cells. Indeed, as we use a pool of PBMC coming from different donors, T cells activate each other by recognition of non-self-antigens present on the surface of the cells coming from the other donors. Activation of T cells leads to their division, similarly to what happens physiologically. Second, it reduces the biological variability by pooling PBMC from ten healthy donors. Third, it can be set up relatively quickly in a quality control laboratory, thanks to the low technicality and little handling time. Indeed, once the CTV-PBMC bank is established, it requires little handling time to test MSC samples with this method. Less than half a day in total is required to set up the co-culture and to proceed to flow cytometry analysis after the 7 days of co-culture. Finally, it has an acceptable cost for a potency assay of a cellular medicinal product. However, it presents some disadvantages and critical points. For instance, 7 days of cell culture are required to obtain sufficient and reproducible T cell PD (data not shown). Moreover, this method does not use a specific population of immune cells as CTV-PBMC bank is composed of lymphocytes, monocytes, and neutrophils (see the “Results” section, bank validation). Finally, we observed a low inter-bank variability of CTV-PBMC. However, each CTV-PBMC bank should be rigorously validated.

We aimed to provide analytical validation data that were not available in the literature to assist MSC production facilities. Therefore, we decided to implement this MLR in our laboratory, according to the ICH Q2 (R1) guidelines.

First of all, we verified that the CTV-PBMC bank, which is critical in this method, was sufficiently stable over time and did not vary when changing the bank. We demonstrated its stability over 509 days, making it easy to use over a long time, consistent with the literature showing storage stabilities of PBMC cryopreserved in liquid nitrogen for several years [12]. Therefore, a CTV-PBMC bank can be used to qualify several productions of MSC batches in the context of one clinical trial. Moreover, in our study, we did not find any differences between the two banks of CTV-PBMC. Investigation of additional banks would be necessary to confirm these results. However, pooling PBMC from 10 donors appeared to have sufficiently reduced the biological inter-variability of T cell PD. It also allows to overcome the influence of the PBMC composition that could usually lead to an increase or a decrease of T cell activation (e.g., monocyte or antigen presentation cell proportion).

The method demonstrated sufficient robustness concerning the number of plated CTV-PBMC but presents a sensibility for MSC variation mostly on co-culture ratio 1/10. Indeed a variation of 6.7% in MSC quantity at this ratio can lead consequently to a modification of the PBMC PD. Thus, we recommend to master and control two factors influencing this MSC quantity: (1) the metrology of the sampling pipettes and (2) the intermediate precision of the MSC count.

The assay exhibits linearity over the 1/10, 1/30, 1/100, and 1/300 BM-MSC/CTV-PBMC ratios, with correlation coefficients close to 1, when considering the curves obtained for repeatability and intermediate precision.

Observed CV for repeatability and reproducibility were 6.1% and 4.6%, respectively, which is less than 10%, the maximum value of variability tolerated for functional, and analytical methods for which there are no published data.

Finally, significant differences were highlighted when applying IFNγ treatment to MSC. This assay can distinguish different ranges of immunomodulatory properties, which proves the ability of this method to discriminate a more or less functional batch of MSC.

One bias in our analytical validation is that we did not perform method comparison and/or reproducibility. Currently, there is no reference or standard method for the qualification of the immunomodulatory capacity of MSC. It is also essential to consider the limitation of the scope of this method. Indeed, it will only apply to the production of a single-center that determines its limits and establishes the final product specifications based on its own MSC production processes and indications. Results of the AUCs should be correlated with the biological and clinical responses of MSC injection in ongoing clinical trials NCT02213705 and NCT03562065 to determine these specifications. Testing this correlation will also help to answer an essential question: does this in vitro assay reflect the immunomodulatory capacity of MSC in vivo and their clinical efficacy?

## Conclusions

To conclude, our validation data obtained according to the ICH Q2(R1) guidelines confirm the results of the team of the Experimental and Clinical Cell Therapy Institute of Salzburg in Austria. However, MSC quantification should be checked before culture to reduce variations. Nonetheless, this MLR has sufficiently analytical parameters to quantify the immunomodulatory activity of MSC on T cells. It can be used in the qualification of the pharmaceutical production of MSC batches.

## Data Availability

All data obtained or analyzed in this study are included in this published article and its supplementary information files. Please contact the author for data requests.

## Abbreviations

AUC: Area under the curve
BM-MSC: Bone marrow-isolated mesenchymal stromal cells
CD: Cluster of differentiation
CTV: Cell trace violet
CTV-PBMC: Cell trace violet-labeled peripheral blood mononuclear cells
CV: Coefficient of variation
DMSO: Dimethyl sulfoxide
EDTA: Ethylenediaminetetraacetic acid
EFS: Établissement Français du Sang
FBS: Fetal bovine serum
FGF: Fibroblast growth factor
GMP: Good Manufacturing Practices
ICH: International Council on Harmonization
IFNγ: Interferon gamma
ISCT: International Society for Cell & Gene Therapy
MSC: Mesenchymal stromal/stem cells
MEM α: Minimum essential medium α
MLR: Mixed lymphocyte reaction
PBMC: Peripheral blood mononuclear cells
PBS: Phosphate-buffered saline
PD: Percentage of division
PI: Percentage of inhibition
RPMI: Roswell Park Memorial Institute
SD: Standard deviation
